# An Unusual Instigator of Spontaneous Bacterial Peritonitis: Salmonella typhimurium

**DOI:** 10.7759/cureus.51960

**Published:** 2024-01-09

**Authors:** Madhulika L Mahashabde, Yash R Bhimani, Gaurav A Chaudhary

**Affiliations:** 1 General Medicine, Dr. D.Y. Patil Medical College, Hospital and Research Centre, Dr. D.Y. Patil Vidyapeeth, Pune, IND

**Keywords:** spontaneous bacterial peritonitis, portal vein thrombosis, hemorrhagic ascitis, unusual case, salmonella typhimurium

## Abstract

*Salmonella* is an unusual cause of spontaneous bacterial peritonitis (SBP). It is commonly seen in asymptomatic patients with normal or high ascitic fluid protein levels and an immunocompromised state such as AIDS and hematological and solid organ malignancies other than liver. SBP from non-typhoidal *Salmonella* species should be considered, even in the absence of underlying immunosuppression. Our patient presented with a history of high-grade fever and frequent loose stools with decompensated alcoholic liver cirrhosis. While evaluating the SBP etiology, ascitic fluid turned out positive for the non-typhoidal *Salmonella* species, which was red, turbid, and hemorrhagic due to portal vein and superior mesenteric vein thrombosis. We thus report an extremely rare case of SBP caused by *Salmonella* *typhimurium* in our patient.

## Introduction

Spontaneous bacterial peritonitis (SBP) is a commonly encountered complication in liver cirrhosis and ascites. The three most common organisms responsible for SBP are *Escherichia* *coli*, *Streptococcus*, and *Klebsiella*. *Salmonella* species is an unusual cause of SBP. *Salmonella typhimurium *is a rod species of the genus *Salmonella. Salmonella typhimurium* is a primary enteric pathogen, usually causing gastroenteritis in humans and other animals. Despite treatment, the SBP mortality is 30% [1]. Therefore, in suspected SBP, prophylactic antibiotics should be started despite low polymorphonuclear neutrophil (PMN) cell count while awaiting an ascitic fluid culture report. Here, we present an unusual case of SBP from non-typhoidal *Salmonella*, complicated with hemorrhagic ascites from an acute portal and superior mesenteric vein thrombosis in an alcoholic liver cirrhosis patient.

## Case presentation

The case was of a 60-year-old man who had alcoholic liver cirrhosis for 12 years and had been hepatitis C positive for six years (probable cause being blood transfusion seven years ago) and was not on any medication for hepatitis C. He was admitted four times within a span of seven years (last admission two years ago) for jaundice, abdominal distension, and upper gastrointestinal (GI) bleeding and had undergone esophageal variceal ligation three times before. The patient was admitted with complaints of yellowish discoloration of urine and sclera for 20 days, abdominal distension and bilateral pedal edema for 15 days, continuous high-grade fever, and mild diffuse abdominal pain for five days. The patient had a personal history of consumption of 180 ml/day of country liquor for the past 15-20 years. He had been abstinent for two months. On general examination, he was vitally stable with the presence of pallor, icterus, and pedal edema. Systemic examination showed a distended abdomen with an abdominal circumference of 101 cm. Shifting dullness was present with moderate ascites and mild splenomegaly. On admission, routine laboratory investigations were as shown in Table 1.

**Table 1:** Values of routine laboratory investigations during first and second admission. SGOT: Serum Glutamic Oxaloacetic Transaminase; SGPT: Serum Glutamic Pyruvic Transaminase; ALP: Alkaline Phosphatase; ANA: Antinuclear Antibody; IF: Immunofluorescence; LKM: Liver-Kidney Microsome; IgM: Immunoglobulin M; IgG: Immunoglobulin G

Parameters (normal limit)	Report (1st admission)	Report (2nd admission)
Hemoglobin (13.2–16.6 gm/dl)	6.3 gm/dl	13.20 gm/dl
Total leucocyte count (4,000–10,000 /µL)	2,200 /µL	9,400 /µL
Platelet count (1,50,000–4,10,000 /µL)	45,000 /µL	30,000 /µL
Serum urea (17–49 mg/dL)	56 mg/dL	90 mg/dL
Serum creatinine (0.6–1.35 mg/dL)	0.67 mg/dL	1.48 mg/dL
Urine routine microscopic examination	15–20 pus cells	Within normal limit
Total serum bilirubin (0.2–1.2 mg/dL)	4.02 mg/dL	3.23 mg/dL
Direct bilirubin (<0.5 mg/dL)	0.43 mg/dL	1.75 mg/dL
Indirect bilirubin (0.1–1 mg/dL)	3.57 mg/dL	1.48 mg/dL
SGOT (8–48 IU/L)	41 IU/L	46 IU/L
SGPT (7–55 IU/L)	23 IU/L	23 IU/L
ALP (40–129 IU/L)	46 IU/L	49 IU/L
Serum albumin (3.5–5 gm/dL)	2.6 gm/dL	2.5 gm/dL
Albumin:globulin ratio	0.68	0.67
D dimer (0–500 ng/mL)	>10,000 ng/mL	7,710 ng/mL
Fibrinogen (180–350 mg/dL)	185 mg/dL	354 mg/dL
Serum procalcitonin (<0.05 ng/mL)	2.84 ng/mL	5.14 ng/mL
Prothrombin time (10.8–13.7 secs)	17.6 secs	18.30 secs
International normalized ratio	1.49	1.55
HIV antibody	Negative	Negative
Hepatitis B antibody	Negative	Negative
Anti-hepatitis C antibody	Positive	Positive
Dengue NS1, IgM, IgG	Negative	Negative
Rapid malarial test	Negative	Negative
ANA by IF	Negative	Not repeated
Anti LKM antibody	Negative	Not repeated
Hepatitis C viral load (<10 IU/ml: Not detected)	<10 IU/ml	Not repeated

Abdominal paracentesis was done, and the results of the ascitic fluid routine microscopy examination are shown in Table 2.

**Table 2:** Diagnostic ascitic fluid routine microscopy values during first and second admission. CB-NAAT: Cartridge Based-Nucleic Acid Amplification Test

Ascitic fluid parameters (normal limit)	Report (1st admission)	Report (2nd admission)
Appearance (straw-colored)	Reddish, turbid	Reddish yellow, turbid
Cobweb (absent)	Absent	Absent
Glucose (>60 mg/dL)	83 mg/dL	<5 mg/dL
Total protein (<3 gm/dL)	4.10 gm/dL	4.20 gm/dL
Albumin	1.7 gm/dL	1.6 gm/dL
Total white blood (0–500/ cmm)	700/cmm	14,400/cmm
Neutrophils	20%	70%
Lymphocyte	65%	15%
Red blood cells (absent)	Many	Moderate
Serum ascitic albumin gradient	0.9	1
Adenosine deaminase (< 39 IU/L)	11.78 IU/L	67.65 IU/L
CB-NAAT and malignant cytology	Negative	Negative

**Figure 1:**
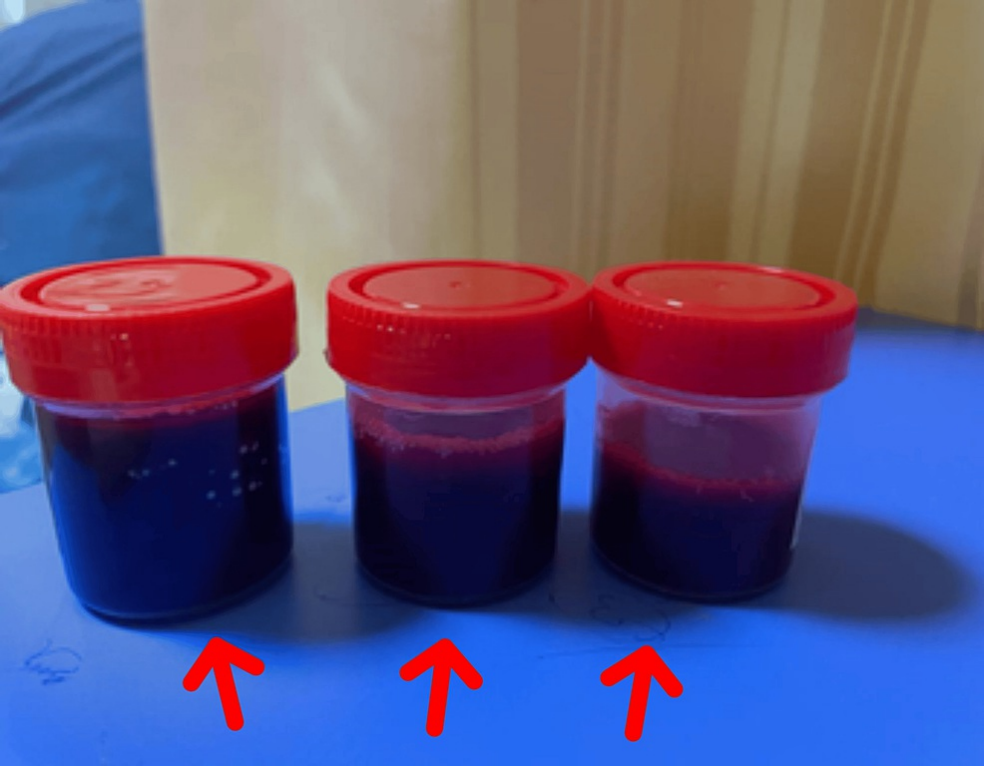
Figure showing reddish, turbid hemorrhagic ascitic fluid (as seen with multiple red arrows).

**Figure 2:**
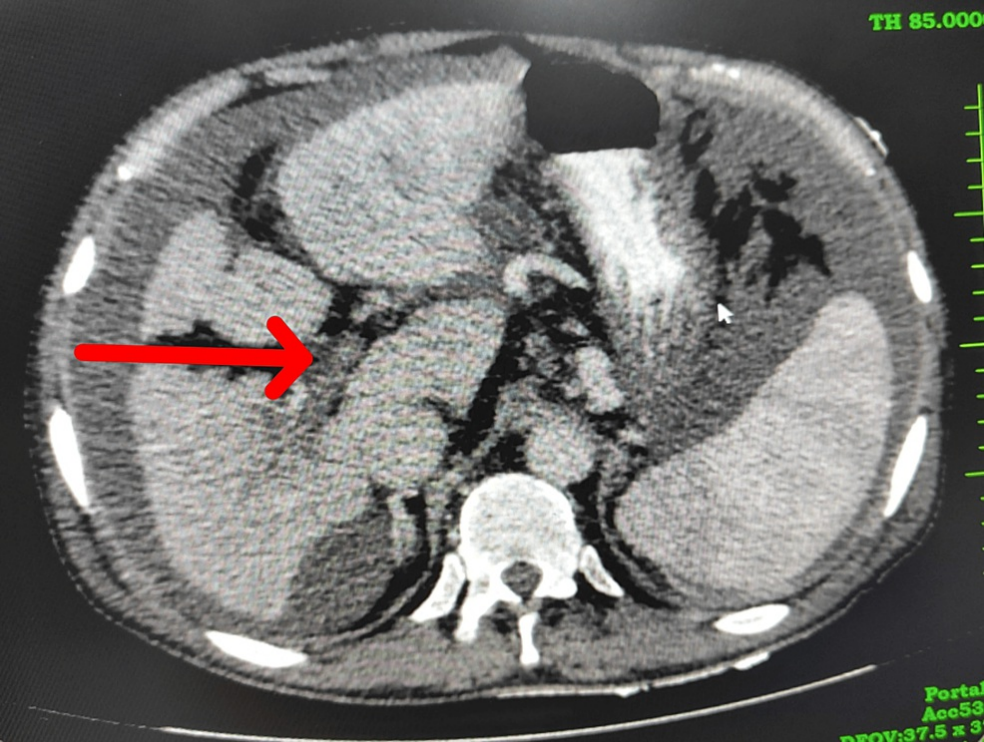
Axial section of contrast-enhanced CT scan of abdomen and pelvis showing portal vein thrombosis and collateral formation (red arrow).

Upper GI endoscopy suggests three columns of large esophageal varices with a red-color sign with portal hypertensive gastropathy. Esophageal band ligation was done. Echocardiography was normal. On day 3 of admission, the patient had breathlessness and was shifted to the ICU. CT scan of the thorax with CT pulmonary angiography showed no evidence of pulmonary thromboembolism. Contrast-enhanced CT abdomen and pelvis showed cirrhosis of the liver with portal hypertension, splenomegaly with portal vein, and inferior mesenteric vein thrombosis with collaterals. Bone marrow aspiration and biopsy suggested mildly hypercellular marrow with mild erythroid hyperplasia with hemophagocytosis with a mild increase in reactive plasma cells with reduced marrow iron stores, suggestive of post-viral changes in a known case of hepatitis C virus infection. HCV RNA report suggested non-detectable viral RNA copies. The ascitic fluid sample was reddish and turbid in appearance (as seen in Figure 1). Contrast-enhanced CT abdomen-pelvis indicated cirrhosis of the liver with portal hypertension, splenomegaly with portal vein, and inferior mesenteric vein thrombosis with collaterals (as seen in Figure 2) and mild-to-moderate ascites. 

**Figure 3:**
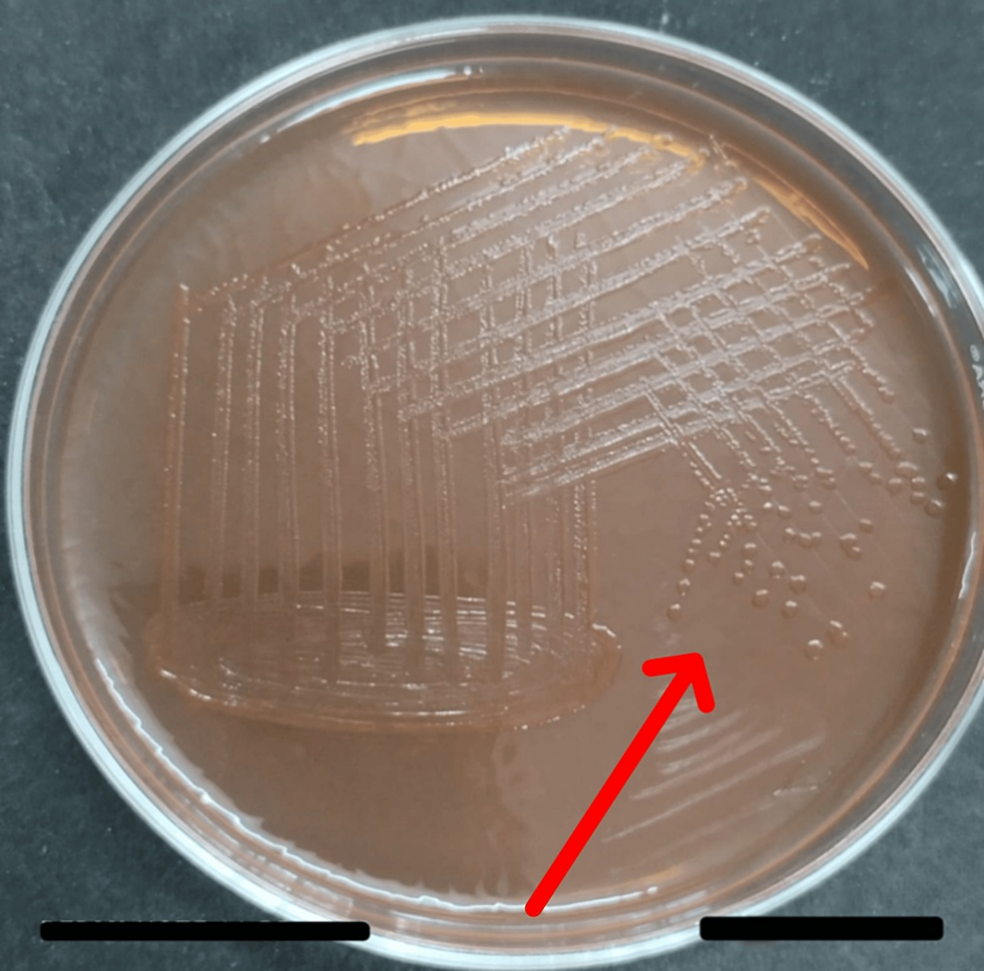
MacConkey agar showing colonies of Salmonella typhimurium (red arrow).

The patient was treated for decompensated liver cirrhosis and was discharged on medication. However, the patient was admitted again after 15 days with complaints of increasing abdominal distension, pedal edema, diffuse abdominal pain for five days, continuous high-grade fever for four days, and 8-10 episodes/day of watery, foul-smelling loose stool without blood for three days. Routine laboratory investigations were as shown in Table 1. Abdominal paracentesis was done and ascitic fluid routine microscopy examination were as shown in Table 2. The ascitic fluid culture was done on MacConkey agar and grew gram-negative bacilli, *Salmonella* *typhimurium* species from the non-typhoidal *Salmonella* group. MacConkey agar was positive for non-*Salmonella* species, *Salmonella* *typhimurium* subtype (as seen in Figure 3). The patient was started on an injection of ceftriaxone IV as an empirical antibiotic. Diuretics, long-acting beta-blockers, and hepatic encephalopathy measures were initiated. IV albumin 20% was given on days 1 and 3. The culture of the ascitic fluid showed *Salmonella* *typhimurium* species, and he was placed on piperacillin-tazobactam 4.5 gm IV eight hourly, as per the sensitivity report. Tablet ciprofloxacin 500 mg once a day was given as prophylaxis for SBP (to be continued indefinitely as patient already has decompensated liver disease) on discharge to prevent secondary bacterial peritonitis. The patient is on regular follow-up without any relapse.

## Discussion

SBP is one of the most commonly found bacterial infections in liver cirrhosis and is detected in 35% of patients admitted to Asian hospitals [2]. SBP recurrence is seen in 44% of patients. Its mortality is around 30% [1]. Organisms responsible for SBP are usually from the Enterobacteriaceae family [3]. The three most common organisms responsible for SBP from 263 ascitic fluid culture reports, compiled in 1994 from different studies published between 1971 and 1991, were *E*. *coli* (46%), *Streptococcus* (30%), and *Klebsiella* (9%) [4]. If more than two organisms are detected, bacterial peritonitis due to secondary causes such as perforated viscus should be considered. SBP is diagnosed when the ascitic fluid has an absolute neutrophil count >250/μL and the ascitic fluid culture is positive. Deficient opsonic activity due to the low-protein content of ascitic fluid predisposes to SBP. High opsonic activity in the ascitic fluid protects from the usual organisms causing SBP unless caused by a highly virulent organism such as *Salmonella* [5]. The immune status and the virulence of the organism play a significant role in SBP occurrence [6]. Sometimes SBP due to *Salmonella* species can be asymptomatic and a low PMN cell count in the ascitic fluid can delay the diagnosis [7]. IV antibiotics should be started empirically in all patients having an ascitic fluid PMN count >250/mm^3^. For community-acquired infection, third-generation cephalosporin is the drug of choice for SBP [4]. For patients with multi-drug-resistant organisms and nosocomial infection, piperacillin/tazobactam 3.375 gm IV six hourly for five days is considered, and in patients with methicillin-resistant *Staphylococcus aureus* (MRSA), vancomycin 15 to 20 mg/kg/dose every 8 to 12 hourly for four to five days is recommended. Prophylaxis should be started in all high-risk patients. Norfloxacin prophylaxis should be started in all patients with liver cirrhosis and low ascitic-fluid protein and also in those with at least one episode of SBP in the past [8]. However in our patient, we started ciprofloxacin based on cultural and sensitivity report. The goal of primary prophylaxis is to prevent SBP occurrence in patients with cirrhosis and ascites and that of secondary prophylaxis is to prevent further episodes of SBP in those with at least one episode of SBP. Venous stasis is commonly observed in liver cirrhosis and can play an important role in the development of portal vein thrombosis which cause development of collaterals which are leaky leading to hemorrhagic ascites. An increase in fibrotic tissue deposition and dynamic intrahepatic resistance can reduce the velocity of portal blood flow, which may predispose to portal vein thrombosis [9]. 

## Conclusions

SBP caused by non-*Salmonella* species is very rare. Normal or low ascitic-fluid PMN cell count can be misleading. However, physicians should keep their differentials wide to identify such rare occurrences. SBP mortality is very high, so a culture should be sent immediately and prophylactic antibiotics should not be delayed. Very high recurrence of SBP emphasizes the importance of early initiation and continuation of primary and secondary prophylaxis.
